# A Dynamic Multi-Mobile Agent Itinerary Planning Approach in Wireless Sensor Networks via Intuitionistic Fuzzy Set

**DOI:** 10.3390/s22208037

**Published:** 2022-10-21

**Authors:** Tariq Alsboui, Richard Hill, Hussain Al-Aqrabi, Hafiz Muhammad Athar Farid, Muhammad Riaz, Shamaila Iram, Hafiz Muhammad Shakeel, Muhammad Hussain

**Affiliations:** 1 Department of Computer Science, School of Computing and Engineering, University of Huddersfield, Queensgate, Huddersfield HD1 3DH, UK; 2 Department of Mathematics, University of the Punjab, Lahore 54590, Pakistan

**Keywords:** wireless sensor networks, mobile agent, itinerary planning, dynamic itinerary, intuitionistic fuzzy set, human–robot interaction

## Abstract

In recent research developments, the application of mobile agents (MAs) has attracted extensive research in wireless sensor networks (WSNs) due to the unique benefits it offers, such as energy conservation, network bandwidth saving, and flexibility of open usage for various WSN applications. The majority of the proposed research ideas on dynamic itinerary planning agent-based algorithms are efficient when dealing with node failure as a result of energy depletion. However, they generate inefficient groups for MAs itineraries, which introduces a delay in broadcasting data return back to the sink node, and they do not consider the expanding size of the MAs during moving towards a sequence of related nodes. In order to rectify these research issues, we propose a new Graph-based Dynamic Multi-Mobile Agent Itinerary Planning approach (GDMIP). GDMIP works with “Directed Acyclic Graph” (DAG) techniques and distributes sensor nodes into various and efficient group-based shortest-identified routes, which cover all nodes in the network using intuitionistic fuzzy sets. MAs are restricted from moving in the predefined path and routes and are responsible for collecting data from the assigned groups. The experimental results of our proposed work show the effectiveness and expediency compared to the published approaches. Therefore, our proposed algorithm is more energy efficient and effective for task delay (time).

## 1. Introduction

The ability to perform fundamental functions by sharing resources within a network and node domains characterises a prevalent network that is interconnected, also expanding to wireless sensor networks (WSN).

WSNs’ main objective is to enable users to acquire relevant information from data collected by spatially distributed sensors. To have complete view of the controlled physical environment, many sensors are typically deployed. Large amounts of data will be created by these sensor network systems, as stated in [1]. Effective collective data collection from (wireless sensor networks) WSNs has been made possible by the widespread deployment of MA techniques. In these methods, MAs are sent out to traverse the sensor network following predetermined routes that are established via itinerary planning in order to collect data from the related nodes along the route. The necessity to find and handle MAs in the energy-efficient systems of WSN, which is essentially defined by methods for designing MA itinerary planning.

Itinerary planning is challenging due to practical restrictions for the deployment of complex nodes and networks, for instance, processing capabilities and the limitation of battery power. The movement cost of a MA, route planning, and the techniques deployed for the designing of such a plan are essential aspects when transmitting MAs to the network.

The MA’s key aim is to collect, use, and process collected data within a network. They can come together and decide locally on their own without user input. Because radio communication is one of the most efficient hungry activities, mobile agents are typically utilised [2]. We, therefore, send agents with the aim of procuring data despite transmitting it towards a sink node to prevent long-distance radio communication. Planning the route of mobile agents in such cases is essential to reducing sensor node energy usage. However, it has proved difficult to tackle the “NP-hard” problem of determining an optimal route fro MAs to visit sensor nodes sequence [3,4].

A significant amount of research has been performed over the past few decades to produce human-like gaze control in an effort to improve natural human–robot interaction (HRI). This endeavour is part of a larger effort to increase HRI [5]. The fundamental idea behind HRI is that the robot should behave in a manner similar to that of an animal friend to a human. According to this paradigm, the robot should neither be shaped to copy the human person nor should it develop communication similar to that which occurs between humans; rather, it should follow the models set by biological systems already in place and form an interaction between different species [6]. This study does not cover systems that use fuzzy logic for sensing, modelling, or planning activities; rather, it only considers systems that use fuzzy logic for inferencing or reasoning tasks. This is because fuzzy logic has already been widely employed in practically all research fields in robotics [7].

The development of an acceptable itinerary for (mobile agents) MAs to gather the related data from all sensors is thus a major task [8]. Planning an itinerary is the process of deciding the path an MA uses when navigating and visiting the network to collect data from the nodes. Each route involves a list of related connected nodes that will stay linked throughout the MA movement journey. The existing techniques can be largely divided into three classifications for establishing MA itineraries: the first is a static itinerary, the second one is a dynamic itinerary, and the third is a hybrid itinerary [8]. In a static itinerary, the dispatcher, or sink node, specifies the order of the nodes to that the mobile agent will pay a visit. In the dynamic itinerary, MAs change the path of the visit on the fly to avoid node failure in the network, whereas, in the hybrid itinerary, the list of sensor nodes to be visited is generated by the sink node, and is changeable on the fly.

Contribution: Our research focuses on the development of a Graph-based Dynamic Multi-Mobile Agent Itinerary Planning approach (GDMIP) that utilises Directed Acyclic Graph (DAG) and intuitionistic fuzzy set in the planning as well as dispatching of mobile agents.

The contributions of this paper are presented as:

• Dynamic multi-mobile agent (MMA) itinerary planning approach for managing resources in a more energy-efficient and scalable manner.
• A novel approach, dubbed GDMIP. GDMIP computes mobile agents itinerary planning based on the “Directed Acyclic Graph” (DAG) technique and selects the shortest route encompassing all the assigned nodes in the proposed network using intuitionistic fuzzy sets. It segments DAG into small groups and allocates mobile agents (MAs) to distinct groups to be more energy efficient.
• In the end, we conducted several experiments to validate the effectiveness and usefulness of the proposed dynamic approach.

The structure of the remaining research paper is presented as follows: Section 2 presents the previous related work providing a segue into our work and contribution. Section 3 describes our methodology. In Section 4, an in depth discussion of the benefits of our framework, along with a comparison with similar and competing methodologies. Lastly, in Section 5, we present our conclusions along with future research directions.

## 2. Related Work

The majority of published work found in the literature assumes that an MA has a lack of prior knowledge of their path when it comes to dynamic itinerary planning in the wireless sensor network for mobile agents. They also employ MA to gather information. A dynamic “Energy and Trust Aware Mobile Agent Migration” (ETMAM), for instance, is suggested in [9]. The primary goal of ETMAM is to locate and avoid hostile or defective nodes when migrating mobile agents. In order to design routes quickly for the travelling agent to carry out the data aggregation activities, ETMAM combines energy and trust as selection criteria. The suggested method is secure, responds to network node failures effectively, and uses little energy. Additionally, ETMAM optimises the agent migration path and lessens the agent payload through the usage of cloning mechanisms. When visiting sensor nodes, ETMAM, however, does not account for the MA’s expanding size. Additionally, it causes a delay in data reporting because the cloned MAs still need to visit a lot of nodes. Furthermore, the entire strategy is regarded as difficult and necessitates considerable processing due to the requirement of recognising malicious nodes.

A dynamic technique of constructing a mobile agent path with a minimal payment based on referral was proposed in [10]. Referral is a cooperative approach for discovering the solution with assistance, presuming that the person asking the question is unaware of the solution but is aware of those who can assist him. Therefore, based on his acquaintance’s knowledge, the present workplace provider can suggest the next workplace (host) for a mobile agent. The suggested method can adapt to an open environment because it is energy-efficient and does not require a pre-fixed system. However, the method lacks security and scalability. Additionally, because it only employs one MA, a delay is introduced.

In [11], a dynamic itinerary planning method based on the Mobile Agent Electronic Triage Tag (MAETT) system is suggested. In order to provide a straightforward system for flexibly defining and tracking an agent’s journey, the aim is to decouple the itinerary data structure from the agent itself. The suggested strategy is scalable and energy efficient. Additionally, it responds effectively when a node fails. However, the method is insecure and requires that the injecting node, which is the one connecting to the portable device, be kept inside the WSN’s connection range. Because of this, medical personnel are required to employ that node as a component of the WSN, replacing the injecting node each time a new WSN is generated. Refs. [10,12] suggests similar strategies. By providing a dynamic planning technique for a mobile agent path based on the Markov decision process (MDP) and social acquaintance recommendation variable decision space in [10], the authors expand on their earlier work. The path planning model is explained with MDP, social acquaintance recommendations, and the assumption that each social member can cooperatively recommend a workplace and service to a mobile agent. The method uses less energy and reacts to node failure quickly. However, it takes a lot of time and effort to actually use their method. Additionally, it causes a delay, lacks security, and is not scalable.

A different strategy is one that is suggested in [13], where the authors focus on the computing side of itinerary planning to let an agent complete its task while adhering to a deadline. A subset of resources that the agent must retrieve from network servers while adhering to a partial order defines the mission. The term “resource” refers to an ambiguous idea that could, among other things, refer to a device, a database, or processing power. The network servers offer resources of various types and quality, so choosing which servers to visit first and in what sequence to do so will help complete the job on time and with the highest quality possible. By establishing a graph-theoretic model for itinerary computation within time constraints, the work presented in [12] advances the work on computation itineraries. This model enables the creation of provably optimum, dynamic programming (DP), and approximation algorithms. These methods respond to node failure effectively and use little energy. They do not, however, offer security support, and the lengthy routes for a particular set of MAs cause a delay due tothe inefficient grouping of sensor nodes.

Dynamic itinerary planning for MAs (DIPMA) is presented in [14]. With an application-oriented methodology, it gathers data from sensor networks. To be more precise, a real-world application of the DIPMA method is the data collection for frost prediction. The DIPMA operates in accordance with two phases, including feature vector design policy and next-hop determination. Instead of delivering an MA across numerous nodes, the design policy of feature vectors swaps vectors between nodes. The concept of choosing the next hop of MA migration using feature vectors is the subject of the next hop choice. The method takes less time to execute and is scalable and energy efficient. However, it only applies to a certain application because searching for an *n*-hop depth results in creating data at a depth that is not optimal. Further, it introduces a delay in reporting data back due to the fact the MAs are required to visit a large number of nodes in each group, and lacks support for security.

The authors of [15] suggest “spawn multi-mobile agent itinerary planning” (SMIP) to greatly minimize the considerable increases in energy expenditure and time required for data collecting. The spawning mobile agent, which enables the essential MA to set off another MA into a single segment, serves as the foundation for this. Based on Bayesian assessments, the proposal determines the number of clusters using x-means methods. When partitioning is finished, the sink node specifies the number of MAs and their travel routes for each partition. Although the method is scalable, it does not account for node failure in the network, which the authors propose as a future area of study.

A three-phased multiple mobile agent itinerary planning method called GIGM-MIP is introduced in [16]. Using the *k*-means algorithm and a set of produced partitions based on geographic information, the network is divided in the first phase. Each partition may contain a number of mobile agents. The second phase counts the number of mobile agents and defines groups of nodes for each agent. The third phase is focused on determining the route that each mobile agent takes while travelling through the cluster of source nodes. Each partition may get a number of mobile agents. The GIGM-MIP technique is scalable and energy-efficient. However, it does not take into consideration node failure in the network, creates a delay, and does not provide security.

In [17], a novel “central location-based MIP” (CL-MIP) framework was developed. The “visiting central location” (VCL) selection algorithm, SIP method, source-grouping algorithm, and its iterative algorithm are the four components that make up the framework. The high source node density is computed using the VCL selection algorithm. Nodes are grouped, and mobile agents are assigned to specific groups via the source-grouping algorithm. To plan the route of mobile agents, the SIP algorithm is used. Finally, the primary goal of the iterative process is to guarantee that all source nodes are assigned to the designated MAs. The CL-MIP method takes into account a cluster-based approach in which the source nodes are deployed across various clusters and grouped geographically. This shows that if the nodes are present, the CL-MIP cannot be employed if the nodes are sparsely deployed.

The authors of [18] suggested a system called MAMS that uses a mobile server and agent to gather data from sensor nodes placed throughout a sensing field. After gathering data, mobile agents independently move between nodes before returning to the mobile server. In order to route mobile agents, the migration process uses a geographic routing strategy. After gathering data, mobile agents locate the mobile server’s current location and head there with the compiled data. The system’s key component is an efficient and perceptive gathering method. However, it introduces a delay due to inefficient grouping, as the mobile agents will have to visit a large number of nodes.

In [19], a new dynamic itinerary planning method is proposed. The three phases that make up SLMADA suggested scheme’s operation are (1) network setup, (2) zone coordinator selection, and (3) MA migration phase, each of which is in charge of a different duty. The strategy is scalable and energy-efficient. Additionally, load-balancing for mobile agent-based data aggregation is accomplished. The performance of the suggested agent migration scheme under cognitive radio sensor networks may be subpar, as the authors note as future work. However, designing a sleep schedule for the nodes that are visited by the mobile agents is crucial, and this could affect how well the proposed agent migration scheme performs. Lengthy MAs routes may cause a delay in reporting data and a lack of support for security.

Jana et al. proposed a “multi-attribute decision making” (MADM) approach using power Dombi aggregation operators (AOs) [20], a dynamical hybrid approach to design the decision-making process [21], and the MADM technique based on complex AOs [22]. Farid and Riaz proposed some generalized Einstein-interactive geometric AOs [23], Prioritized Interactive AOs [24] for q-rung orthopair fuzzy (q-ROF) information. Riaz et al. proposed prioritized AOs based on priority degrees for q-ROF data [25]. There is some brilliant work related to MADM seen in [26,27,28]. Ashraf et al. introduced some AOs for cubic picture fuzzy sets [29], symmetric sum-based AOs for spherical fuzzy data [30]. Ali et al. gave the notion of complex intuitionistic fuzzy soft AOs [31]. Novel score functions related to q-ROF data are given by Feng et al. [32]. Alcantud et al. [33] proposed a MADM method based on N-soft set. Deveci et al. [34] proposed a Dombi-Bonferroni Operator-based decision-making model.

The authors of [35] introduce FuMAM, a fuzzy-based MA migration strategy, in their approach. By taking into account three factors, remaining energy, distance, and the number of neighbours, FuMAM determines the best itineraries for MA. The proposed use of the FuMAM approach results in an increase in the percentage of successful MA round trips. In addition, the FuMAM algorithm extends the amount of time a network can function by selecting the node in the network that has the highest amount of residual energy as the next hop for MA migration. However, owing to the lengthy MA itineraries, there is a delay introduced.

The FuMAM-based approach is similar to the GDMIP approach proposed in this paper. Both approaches utilise fuzzy logic. However, our approach focuses on intuitionistic fuzzy sets, and we consider different criteria for the MA itinerary, such as grouping, energy efficiency, least number of nodes, and avoiding excessive delay. These criteria are considered efficient in determining the itinerary planning of each MA.

## 3. Proposed GDMIP

### 3.1. GDMIP Architecture

**Figure 1:**
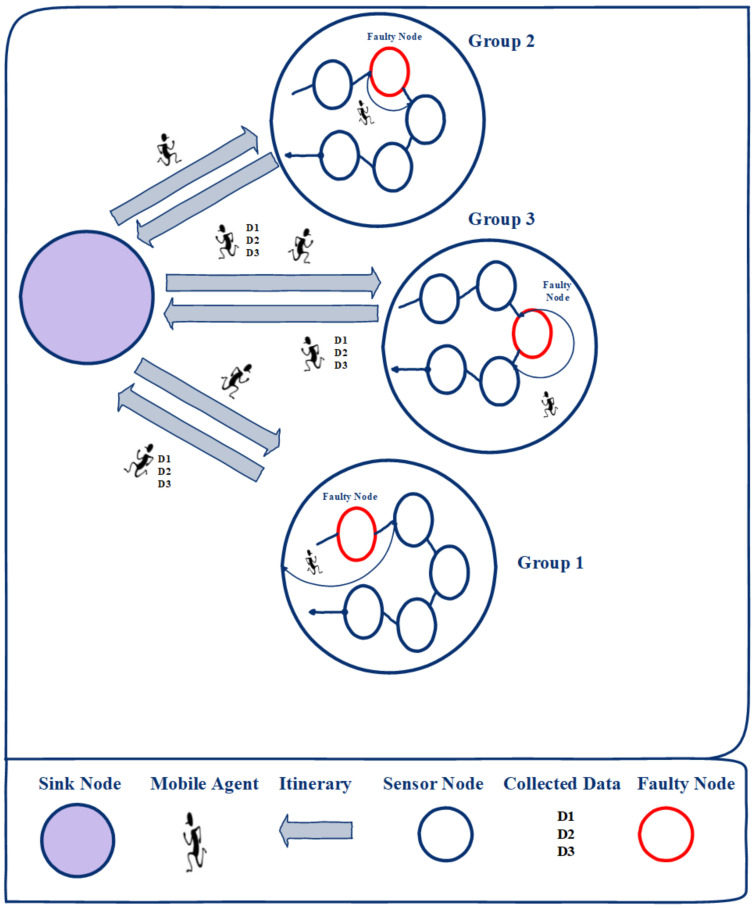
The Proposed Dynamic Approach for Multi-Mobile Agents Itinerary Planning.

The thematic demonstration of our proposed GDMIP approach is shown in Figure 1 (GDMIP). All important elements are described, including itineraries, MAs, nodes, faulty nodes, and gathered data, and each component works its own certain function. It displays the arrangement of the related nodes with the specified route. The discovered shared nodes form the basis of the grouping. All network nodes are covered by the routes that are created. The sink node is in charge of allocating MAs to a specific group to gather data from. If a node is faulty in the group as a result of energy depletion during MA migration, the MA will pass that node in the group. The MA moves and collects data only from the groups that have been assigned to it. As an illustration, the path to the designated group is shown by the “itinerary” (orange line circles).

We employ a number of MAs to prevent reporting delays and to promote local interactions. We use a methodology similar to that described in [36,37] to determine the volume of sensory data that MAs should gather. In order to eliminate duplication and inconsistency, we use equivalent data aggregation techniques. A combination of sensory data effects and the ratio of an aggregation (“ρ,0⩽ρ⩽1”) is used.

$L_{ma}^{i}$ is the quantity of the related sensor data for aggregation via the fusion factor rho, with $A_{i}$ having the actual quantity of related sensor data after the mobile agent receives data input from the source node *i*.

$$L_{ma}^{i}=A_{i}$$

(1) $$L_{ma}^{2}=A_{i}+\left(1-\rho \right)\times A_{2}$$

(2) $$L_{ma}^{i}=L_{ma}^{i}+\left(1-\rho \right)\times A_{2}$$

(3) $$=A_{1}+\sum_{g=2}^{1}\left(1-\rho \right)\times A_{g}$$

Equation (3) indicates that there is no data aggregation for the first node; also the value of ρ depends on the type of application being used.

**Figure 2:**
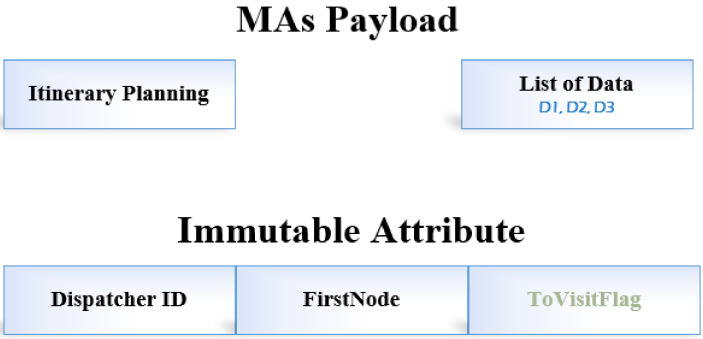
Proposed itinerary message format.

Figure 2 defines the proposed GDMIP data packet format. The initial data collection from the source node and the MA are represented by *FirstNode*. We have established groups and routes. A group merely decides which nodes the MAs should collect data from. However, all computed routes are constructed with MAs migrating to every node on the route.

The agents’ payload consists of the messages *Itinerary Planning* and *List of data*. The node at the root that posts and generates the current mobile agent is recognized by the *Dispatcher ID*. *ToVisitFlag* is set up to check whether an agent visited the node or not. In order to gather data from the nodes along this specific path, routes are computed for the mobile agents to travel towards and visit each node and the group of nodes.

Additionally, we are of the view that sensors are expected to generate similar data when placed close together in order to simulate a real-world scenario. In light of this, agents may remove unnecessary data through fusion.

### 3.2. GDMIP Algorithms

An explanation of the algorithms utilised in the suggested GDMIP approach is provided in this section.

For the purpose of constructing simulation situations, Algorithm 1 is supplied with the pseudocode to produce an arbitrary DAG *G*. Algorithm 2 provides pseudocode for computing routes and generating groups of nodes. Algorithm 3 presents the pseudocode of multi-mobile agent dispatched to start sensory data collection. The multi-mobile agent’s dispatched pseudocode is shown in Algorithm 4, which was used to commence the collection of sensory data.

**Algorithm 1:** Generate a random directed acyclic graph *G*

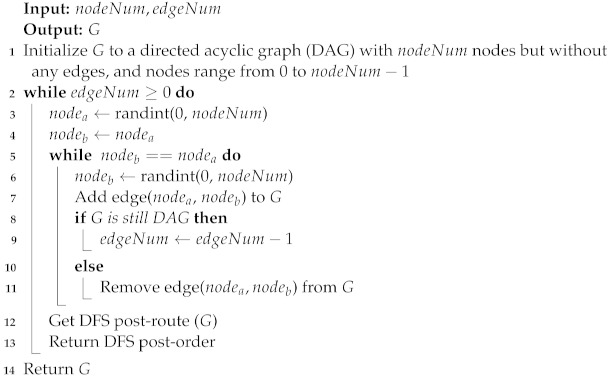



First, we create a random-sized network with a random distribution of nodes and edges using a random DAG, as described in Algorithm 1. The required amount of nodeNum nodes is added by the iterated method. The *G* graph is then positioned to examine if it is completely linked with DAG—that is, if all the related source nodes are moved back after visiting the sink node. A deep first search is used to check and confirm if the relevant nodes have examined.

Communication links between nodes in a typical WSN deployment might be either symmetric or asymmetric. Because it will make our method more flexible, we have presumed asymmetric communication and network links on this occasion. If a communication channel is symmetric, our method can still be used by employing only that one link.

### 3.3. Intuitionistic Fuzzy Weighted Averaging (IFWA)

In multi-mobile agent itinerary planning algorithms, there are many factors involved. These factors are uncertain due to their nature. Fuzzy logic is similar to the natural decision-making process. It interacts with unclear and ambiguous knowledge. This is a simplified view of authentic situations, relying on stages of truth instead of the typical 1/0 or true/false as Boolean reasoning.

The phrase “fuzzy” refers to things that are unclear. We commonly meet circumstances in the real world in which we are unable to determine if such a condition is factually true, such as in this proposed approach, we are uncertain of the identified shortest route with the least number of nodes, in which fuzzy logic provides a highly useful variety of thinking. We may use this strategy to investigate the mistakes and inconsistencies in any circumstance. The Boolean paradigm represents 1.0 as the ultimate truth value and 0.0 as the ultimate false value. However, in the case of fuzzy systems, there is an intermediary value that is both partially true and most likely partially false. Lofti A. Zadeh invented fuzzy logic in his academic research “fuzzy sets (FSs)” in 1965 [38].

A FS *F* in the universe X can be characterised as a set of ordered pairs and formally depicted as

$$F=\{\left(x,\mu_{F}\left(x\right)\right)|x\in X\}$$

here, $\mu_{F}\left(x\right)$ represents the “membership function (MF)” whose range is [0,1].

Fuzzy logics have various uses, such as altitude management of spaceships and rockets in the aviation industry. It has been utilised in automobile systems to manage speed and traffic, for decision-making assistance systems and security appraisal, for chemical process industries to manage the pH, drying, and chemical distillation processes, for natural language translation, and a variety of intense machine learning and artificial intelligence. Fuzzy logic is often employed in control design systems such as expert systems and in conjunction with Neural Networks because it simulates how humans make judgments, only much faster.

It is not always the case that the grade of non-membership of an object in an FS is equivalent to 1 minus the membership degree; this statement is not always accurate. As a result, intuitionistic fuzzy sets (IFSs) were proposed as a generalisation of FSs. The idea of defining IFSs as generalised FSs is intriguing and beneficial in a variety of application areas. Because it incorporates the “degree of belongingness”, “degree of non-belongingness”, and the hesitation margin, the information and semantic description of IFS become more significant, resourceful, and useful. A IFS *I* in the universe X can be characterised as a set and formally depicted as,

$$I=\{\left(x,\mu_{I}\left(x\right),\nu_{I}\left(x\right)\right)|x\in X\}$$

here $\mu_{I}\left(x\right)$ represents the MF and $\nu_{I}\left(x\right)$ represents the non-MF; their ranges are [0,1], with the constraint that the $\mu_{I}\left(x\right)+\nu_{I}\left(x\right))\leq 1,\forall x\in$*X* [39].

Data aggregation is a key step in the selection process across many domains, including corporate, legislative, social, academic, technological, intellectual, and intelligent machines. Throughout the course of human history, comprehension of options has traditionally been regarded as a discrete or spoken amount. In the most recent decades, there has been a significant focus placed on the consolidation of various types of information. The efficiency of the AO, as well as their constraints, have been ingrained in the decision-making process. It should come as no surprise that AO incorporates a variety of standard procedures for combining a limited number of fuzzy numbers into a single fuzzy number.

**Figure 3:**
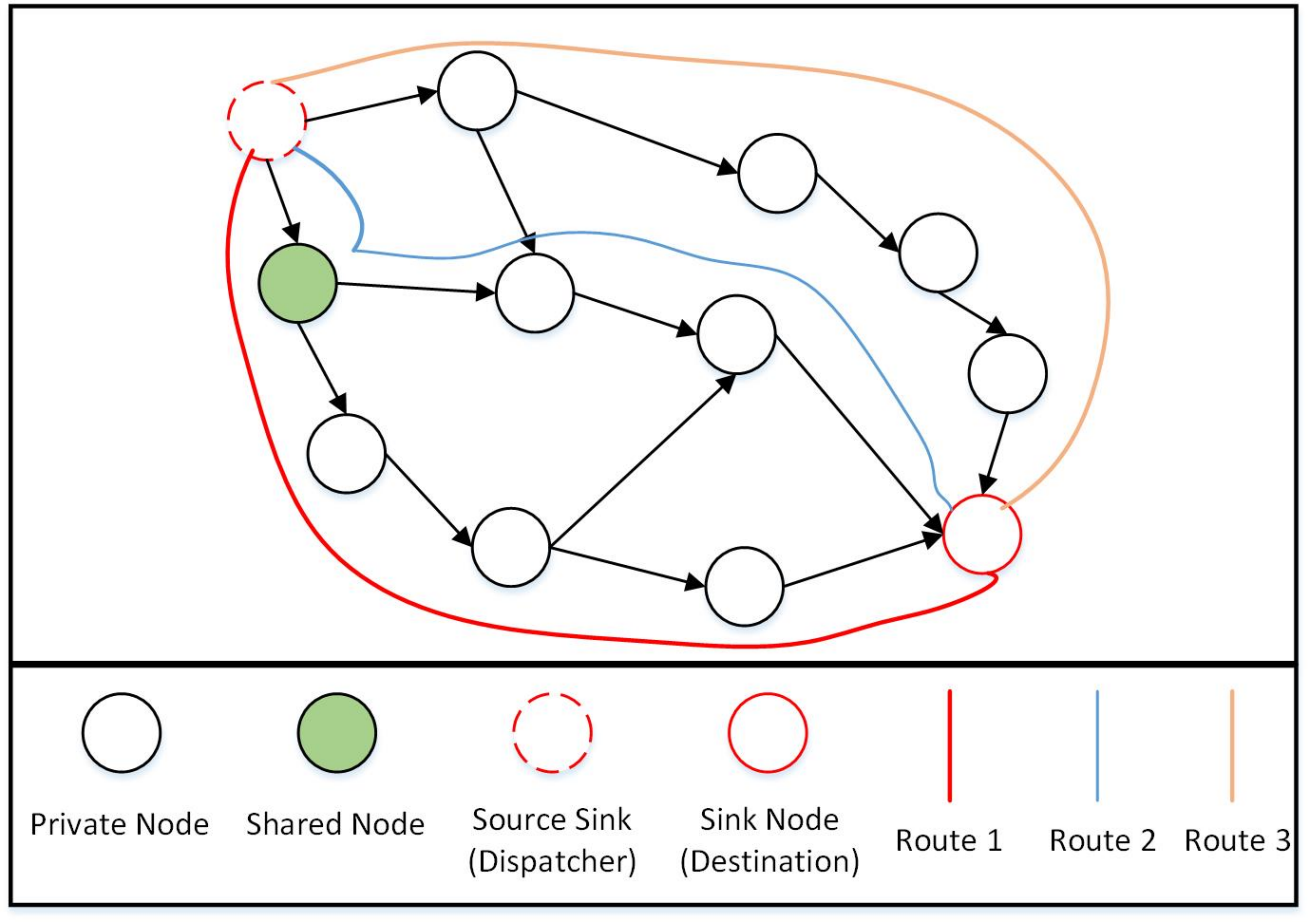
Principle and Working flow of the Algorithms.

Many elements are included in itinerary planning algorithms, including $\left(C_{1}\right)$ grouping, $\left(C_{2}\right)$ energy efficiency, $\left(C_{3}\right)$ least number of nodes, and $\left(C_{4}\right)$ avoiding excessive delays. Because these characteristics are ambiguous and unpredictable, we employ intuitionistic fuzzy numbers (IFNs) to better explain the information. In Figure 3, as can be seen, there are three different routes (route 1, route 2, and route 3), and if we have a decision-maker (DM) to determine the best route based on the parameters (criteria) listed above, the DM assigns an IFN to each route based on its assessments of each criterion. We get a decision matrix after evaluating all paths and then use the “intuitionistic fuzzy weighted averaging (IFWA) operator” to aggregate the data [40]. In IFWA operators, each criterion has a weight vector (WV) that specifies its relevance. An IFWA operator defines if we have a collection of IFNs given as $\alpha_{k}=\left(\mu_{k},\nu_{k}\right)$, where k=1,2,3,…,n. The IFWA operator is a mapping from $X^{n}\to X$ and is given as

$$IFWA\left(\alpha_{1},\alpha_{2},\ldots ,\alpha_{n}\right)=\left(1-\prod_{i=1}^{n}{\left(1-\mu_{\alpha_{i}}\right)}^{\omega_{i}},\prod_{i=1}^{n}\nu_{\alpha_{i}}^{\omega_{i}}\right)$$

where $\omega =(\omega_{1},\omega_{2},\ldots ,\omega_{n})$ is the WV of $\alpha_{i}$ with the constraint that the sum of WV is a unit.

A score function is used to define how one IFN is larger than another IFN. An IFN α having a larger score value than another IFN β, then α is larger than β. The score function is defined as $S\left(\alpha \right)=\mu_{\alpha }-\nu_{\beta }$.

A DM (or group of DMs) evaluates the three routes (route 1, route 2, and route 3) on the basis of the four criteria, $\left(C_{1}\right)$ grouping, $\left(C_{2}\right)$ energy efficiency, $\left(C_{3}\right)$ least number of nodes, and $\left(C_{4}\right)$ avoiding excessive delays, given in Table 1. The WV of these criteria is (0.20,0.25,0.25,0.30). Using the IFWA operator, we aggregate all IFNs for each route to obtain a single IFN for each route. Following that, we apply the scoring function to each aggregated IFN. If an aggregated IFN has a higher score value, the route associated with this IFN is a shorter route. As per the data given in Table 1, the best route is “route 2”. Algorithm 2 is given for the working of an IFWA operator for selecting the optimum route solution.

**Algorithm 2:** Working of IFWA operator to select optimal route

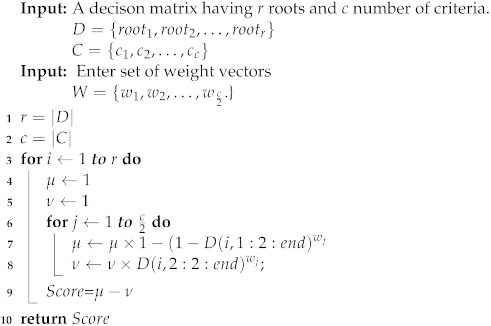



The algorithm related to the source grouping of the input nodes (Algorithm 3) is made explicitly for making a group of the input nodes for all of the MAs, networks, and practices, using *G* from Algorithm 1 as an input. It determines whether the node has two or more out-degree connections and at least one connection or more in-degree connections. This is a shared node as a result. Because of its multiple routes and status as a generating group construction piece, it plays a significant part in the network. It also serves as the primary hub for linking together the nodes in the DAG network. By figuring out the roots and leaves of the network, the specific algorithm learns and determines a set of various routes that cover all the related nodes and paths. Then, it determines every path between the leaves and roots. The source grouping method evaluates all routes to determine which has the fewest nodes based on Algorithm 2, which employs fuzzy aggregation operators, in order to determine the least route. A shared node is one that is a part of numerous routes, according to our definition. This indicates that many routes can be used to access common nodes. Following is how shared nodes between various routes will be distributed: (1) A collection of nodes that are exclusive to a given route are initially assigned to each route (these nodes are referred to as private nodes on the route) and (2) the group that currently has the fewest nodes among the associated routes will be given a shared node. Each created group specifies a collection of nodes from which the sent MAs can gather information.

**Algorithm 3:** Source grouping of sensor nodes

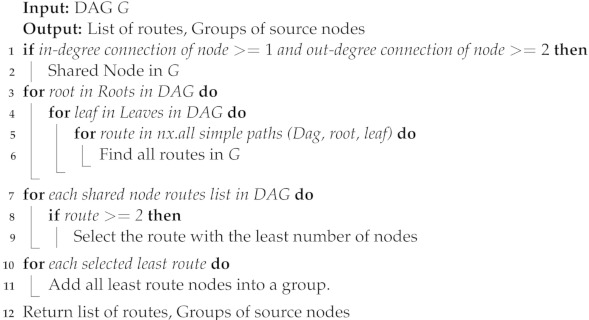



Figure 3 is an illustration of how the algorithms’ underlying ideas work. It displays a series of routes (in this example, routes 1, 2, and 3) that cover all nodes in the network, including shared and private nodes. Private nodes are those that are exclusive to a given route. Source nodes on several routes are referred to as shared nodes. In this example, there is just one shared node, and it is present on both routes 1 and 2. A group is also a collection of assigned shared nodes and private nodes in a specific route. The groups are created by assigning shared nodes to the group of a route that contains the fewest nodes overall. As an illustration, see Figure 3. We assign the shared node to the group for route 2 since there are only two private nodes in the group for route 2, compared to the three private nodes in the group for route 1. Keep in mind that, in practice, the source sink (such as the dispatcher) and the sink node (such as the destination) may be the same (sink) node. Here, since the entire network is modelled as a DAG, it is effectively split into two nodes that each utilise a portion of the network’s links to source nodes.

Mobile agents are dispatched to groups by Algorithm 4. The first inputs are a DAG (G),alistofroutes(R) (r),andasetofsourcenodes(gs), which are provided using Algorithm 3. *T* will then be initialised with a blank bundle of MA data. In order to avoid having two agents take the same group, it starts by sending mobile agents to a certain group in gs. During the journey, each MA visits nodes on the specified group gs. First, it determines whether the node is accessible and online; if so, it gathers the information and marks the node as visited. Then, it checks if the node is offline; if so, it will be marked as dead, and the agent will move to the next node in the group. If the node’s flag visited is set to true, it then moves on to visit the next node in the path. If not, the MA collects data up until its *d* data load and sets this node’s visited flag to true. The MA completes the tasks assigned to it, then leaves by either travelling to every online node along the predetermined route or by gathering *d* data on the trip. The *d* data load threshold for each agent ensures that the agent buffer will not become overloaded with data in a single trip.

**Algorithm 4:** Dispatch mobile agents *MAs* to collect data

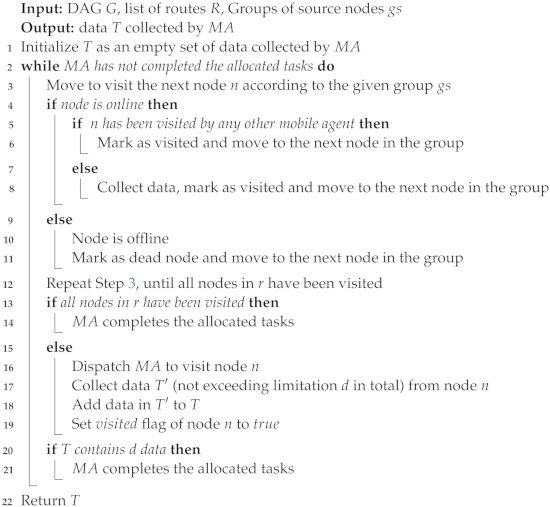



## 4. Experiments, Evaluation and Analysis of Results

### 4.1. Simulation Setup

Using python-based simulation, we implemented and evaluated the proposed GDMIP approach as well as current approaches from the literature, including the SMIP [15] approach, GIGM-MIP [16] approach, and CL-MIP [17]. The network model is taken from [17], where 100 sensor nodes are distributed evenly over a space, and a sink node is positioned in the middle. All of the mobile agent parameters used in the simulation are listed in Table 2.

### 4.2. Simulation Parameters

The sensor nodes are static and evenly distributed over a network with a size of 1000 by 500 m. Where 100 predetermined number of sensor nodes, and they are dispersed randomly throughout the network. The sink node, which is in the middle of the network, has the greater processing power and an endless supply of energy. The starting energy is the same for all of the sensor nodes. The raw data size is *2048 bits*, and the radio transmission range is *60 m*. The raw data reduction ratio is *0.8*, the mobile agent code size is *1024 bits*, the data processing rate is *50 Mbps*, and the mobile agent accessing latency is *10 ms*. The simulations of the trials take into account all sorts of energy usage, including transmission, idling, and sensing. All of the mobile agent parameters used in the simulation are listed in Table 2.

### 4.3. Evaluation and Analysis of Results

The following criteria were used to assess how well various approaches performed: Task Duration and Energy Efficiency are two performance measures that are taken into account. The two performance metrics are defined in Table 3.

**Figure 4:**
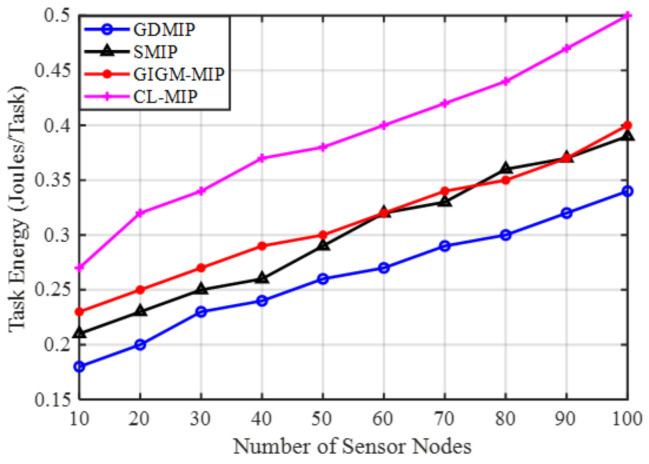
The Impact of Number of Sensor Nodes on Energy Consumption.

Energy Efficiency: The proposed GDMIP approach clearly outperforms SMIP, GIGM-MIP, and CL-MIP approaches in terms of energy utilization, as illustrated in Figure 4. In all four strategies, more energy is needed to execute tasks with more agents. However, it can be seen that the proposed GDMIP saves more energy than other methods. When the number of nodes is reduced from 100 to 10, the proposed GDMIP approach achieves a 32.3% and 14.3% energy reduction compared to SMIP 37.3% and 16.2%, GIGM-MIP 46.4% and 18.2%, and CL-MIP 47.5% and 26.6%. The CL-MIP algorithm uses the most energy. This is due to an increase in the number of mobile agent hops caused by the spread of many mobile agents throughout the sensor network. When the quantity of sensor nodes rises, the GIGM-MIP algorithm uses less energy than the CL-MIP. This is because the GIGM-MIP algorithm divides up the data collected across mobile agents in each network zone. When compared to SMIP, GIGM-MIP, and CL-MIP, the suggested GDMIP consumes less energy. This accomplishment was made possible by effective partitioning, or the grouping approach, as well as the mobile agents’ minimum travel distances inside each group. Fewer trips are required for the mobile agents in each group due to the effective segmentation of the network constructions.

**Figure 5:**
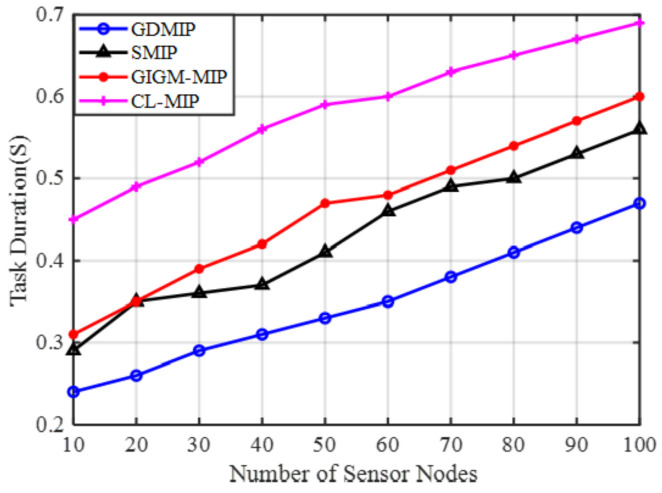
The Impact of the Number of Sensor Nodes on Task Duration.

Task Duration: In terms of task duration, Figure 5 compares the four methods, and it has been found that the proposed GDMIP approach performs better than the three currently used approaches, SMIP, GIGM-MIP, and CL-MIP. The proposed GDMIP delivers the best task duration of all techniques, as can be shown from the observations. The proposed GDMIP reduces task duration by 51.8% and 21.4%, compared to SMIP 55.7% and 29.7%, GIGM-MIP 57.3% and 33.5%, and CL-MIP 66.9% and 41.2%, which has the biggest delay when the number of nodes falls from 100 to 10. Each mobile agent is scheduled to visit all sensor nodes according to the defined routes chosen by the sink node in the SMIP, GIGM-MIP, and CL-MIP algorithms in order to gather data from sensor nodes. There are significant delays as a result of the increased number of mobile agent hops. The proposed GDMIP, however, offers shorter routes and better task duration because fewer nodes must be visited by the mobile agents.

### 4.4. Varying the number of Dispatched MAs

**Figure 6:**
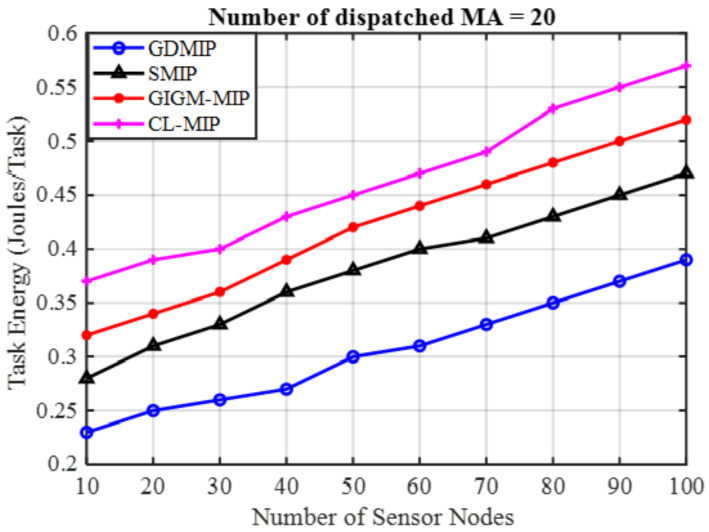
The Impact of Number of Dispatched MAs on Energy Consumption.

**Figure 7:**
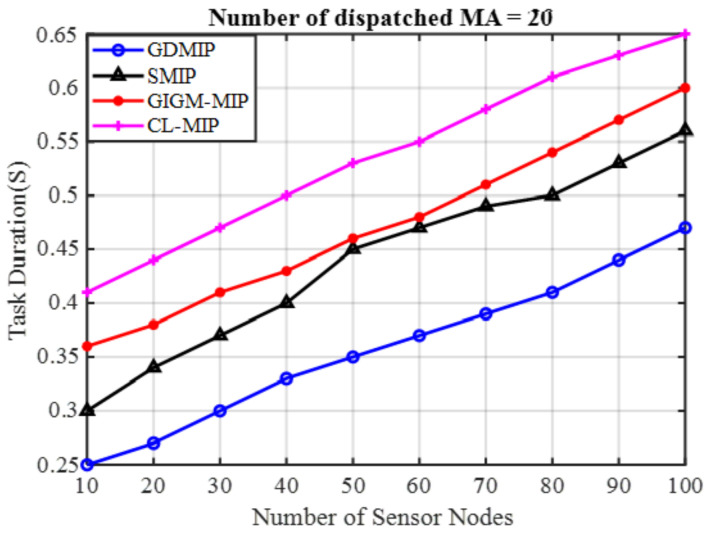
The Impact of the Number of Dispatched MAs on Task Duration.

Figure 6 and Figure 7 show the results of the energy consumption and task duration when the number of dispatched MAs is 20.

Energy Efficiency: As can be seen in Figure 6, which illustrates how the density of the dispatched MAs affects energy usage, it is evident that the suggested GDMIP approach uses 20 MAs less than SMIP, GIGM-MIP, and CL-MIP approaches, while outperforming them in terms of energy consumption. It is evident that the suggested GDMIP saves more energy than alternative methods. When the number of nodes is reduced from 100 to 10, the suggested GDMIP approach delivers energy reductions of 42.4% and 26.2% compared to SMIP 51.6% and 33.3%, GIGM-MIP 58.5% and 35.2%, and CL-MIP% 61.6 and 41.8%. The reduction in the number of hops for each MA within the groups is what allows the GDMIP technique to reduce energy consumption.

Task Duration: as can be seen in Figure 7, which evaluates the four methods in terms of task duration, this experiment dispatches only 20 MAs. It is evident that the GDMIP method was a better performer compared to the other approaches that included not only SMIP but also GIGM-MIP and CL-MIP. It is evident that the GDMIP achieved the optimal task duration compared to the other methods. GDMIP achieved 51.3% and 27.5% task duration reductions in contrast to SMIP 57.6% and 35.8%, GIGM-MIP 64.3% and 38.4%, and CL-MIP 68.3% and 45.4%, having the highest delay when the number of nodes dropped from 100 to 10. This improvement in the GDMIP approach is because a short itinerary is built for each MA in each group.

## 5. Conclusions and Future Work

The multiple MAs approach enhances the data collection capacity of WSNs. The main aim of this research is to develop a dynamic multi-mobile agent itinerary planning approach called GDMIP, where fault tolerance, such as node failure as a result of energy depletion, is taken into consideration. The proposed GDMIP applies an intuitionistic fuzzy set using four criteria, e.g., grouping, energy efficiency, and least number of nodes, to select the shortest route for each dispatched MA. The advantages of the proposed GDMIP include energy efficiency, requiring less delay, and avoiding faulty nodes in the network. The simulation has been carried out to evaluate the performance of the proposed GDMIP. The results indicate that GDMIP has achieved significant improvements in terms of energy consumption and task delay.

There are several interesting directions for future work. We plan to include security by design to protect the GDMIP approach. The security part will focus on selecting a lightweight encryption technique to encrypt the data collected by each mobile agent and protect the overall itinerary planning from any attack. We also plan to make the shared node a leader node, which manages a set of nodes and will be responsible for receiving data from these nodes. Consequently, an MA will be dispatched to collect data from the leader node rather than all of the nodes in each group. This would result in a significant reduction in task duration. The proposed work can be extended to the fuzzy integral-based control of a robotic head for human–robot interaction.

## Figures and Tables

**Table 1:** Decision matrix acquired from DM.

	$\left(C_{1}\right)$	$\left(C_{2}\right)$	$\left(C_{3}\right)$	$\left(C_{4}\right)$
route 1	(0.70, 0.20)	(0.85, 0.10)	(0.75, 0.20)	(0.55, 0.15)
route 2	(0.45, 0.30)	(0.50, 0.20)	(0.65, 0.15)	(0.30, 0.55)
route 3	(0.35, 0.15)	(0.55, 0.25)	(0.25, 0.45)	(0.10, 0.15)

**Table 2:** Simulated Parameters for the Proposed GDMIP Approach.

Network Size	1000 m × 500 m
Sensor Nodes	100
Data Size (raw)	2048 bits
Code Size (MA)	1024 bits
Data Processing Rate	50 Mbps
Data Reduction Ratio (raw)	0.8
Aggregated Ratio	0.9
Transmission Range	60 m
MA delay accessing	10 ms

**Table 3:** Performance metrics for experimental work.

Performance Metrics	
**Evaluation Metrics**	**Definition**
Energy Consumption	refers to the energy used by mobile agents to send and receive messages from all sensor nodes.
Task Duration	refers to the period of time, on average, from when the sink sends out the mobile agents to when the last one returns to the sink.

## Data Availability

Not applicable.
